# The Role of Cytochromes P450 and Aldo-Keto Reductases in Prognosis of Breast Carcinoma Patients

**DOI:** 10.1097/MD.0000000000000255

**Published:** 2014-12-02

**Authors:** Viktor Hlaváč, Veronika Brynychová, Radka Václavíková, Marie Ehrlichová, David Vrána, Václav Pecha, Markéta Trnková, Roman Kodet, Marcela Mrhalová, Kateřina Kubáčková, Jiří Gatěk, Petr Vážan, Pavel Souček

**Affiliations:** From the Toxicogenomics Unit (VH, VB, RV, ME, DV, PS), National Institute of Public Health; 3rd Faculty of Medicine (VH, VB, ME), Charles University, Prague; Department of Oncology (DV), Palacky University Medical School and Teaching Hospital, Olomouc; Institute for the Care for Mother and Child (VP); Biolab Praha, Ltd (MT); Department of Pathology and Molecular Medicine (RK, MM); Department of Oncology (KK), University Hospital Motol, Prague; Department of Surgery (JG), Hospital Atlas; Tomas Bata University (JG); and Department of Pathology (PV), VELAB Ltd, Zlin, Czech Republic.

## Abstract

Metabolism of anticancer drugs affects their antitumor effects. This study has investigated the associations of gene expression of enzymes metabolizing anticancer drugs with therapy response and survival of breast carcinoma patients.

Gene expression of 13 aldo-keto reductases (AKRs), carbonyl reductase 1, and 10 cytochromes P450 (CYPs) was assessed using quantitative real-time polymerase chain reaction in tumors and paired adjacent nonneoplastic tissues from 68 posttreatment breast carcinoma patients. Eleven candidate genes were then evaluated in an independent series of 50 pretreatment patients. Protein expression of the most significant genes was confirmed by immunoblotting.

AKR1A1 was significantly overexpressed and AKR1C1–4, KCNAB1, CYP2C19, CYP3A4, and CYP3A5 downregulated in tumors compared with control nonneoplastic tissues after correction for multiple testing. Significant association of CYP2B6 transcript levels in tumors with expression of hormonal receptors was found in the posttreatment set and replicated in the pretreatment set of patients. Significantly higher intratumoral levels of AKR1C1, AKR1C2, or CYP2W1 were found in responders to neoadjuvant chemotherapy compared with nonresponders. Patients with high AKR7A3 or CYP2B6 levels in the pretreatment set had significantly longer disease-free survival than patients with low levels. Protein products of AKR1C1, AKR1C2, AKR7A3, CYP3A4, and carbonyl reductase (CBR1) were found in tumors and those of AKR1C1, AKR7A3, and CBR1 correlated with their transcript levels. Small interfering RNA-directed knockdown of AKR1C2 or vector-mediated upregulation of CYP3A4 in MDA-MB-231 model cell line had no effect on cell proliferation after paclitaxel treatment in vitro.

Prognostic and predictive roles of drug-metabolizing enzymes strikingly differ between posttreatment and pretreatment breast carcinoma patients. Mechanisms of action of AKR1C2, AKR7A3, CYP2B6, CYP3A4, and CBR1 should continue to be further followed in breast carcinoma patients and models.

## Abstract 2

Supplemental Digital Content is available in the text

## INTRODUCTION

Breast carcinoma is the most common cancer in women worldwide.^1^ The prognosis of breast carcinoma patients vastly depends on the response of the tumor cells to chemotherapy. Decreased uptake or eventually increased efflux of drugs, increased DNA repair or reduced apoptosis, and inactivation of anticancer drugs by biotransformation enzymes may contribute to the development of multidrug resistance.^2^

Phase I biotransformations typically involve substrate oxidation by the cytochrome P450 (CYP) monooxygenases. About 20 enzymes from 57 known CYPs are active in metabolism of procarcinogens and drugs. Most of them lack important functional polymorphisms, but *CYP2A6*, *CYP2B6*, *CYP2C9*, *CYP2C19*, and *CYP2D6* are highly polymorphic suggesting a potential effect on their expression.^3^

Cyclophosphamide is a prodrug that must undergo activation by CYP2C9, CYP2C19, CYP3A4, and CYP3A5.^4,5^ CYP2B6 also participates in cyclophosphamide activation in the liver, but its role in the response to cyclophosphamide in cancer patients has not been proven yet.^6^ CYP2C8, CYP3A4, and CYP3A5 are major taxane-metabolizing enzymes.^7,8^ Roles of CYP1A2, CYP2A6, and CYP2C8 in 5-fluorouracil formation from a prodrug tegafur have been described as well.^9^*CYP2C19* and *CYP2D6* polymorphisms have recently been associated with therapeutic outcome of tamoxifen-treated breast carcinoma patients.^10^

CYP2C9, CYP2D6, and CYP3A4 mRNA expression has unambiguously been detected in mammary gland.^11,12^ Strong protein expression of CYP2S1 and CYP3A4 has been associated with shorter survival time of breast carcinoma patients.^13^ Despite the knowledge about CYP2W1 substrate specificity is limited,^14^ its overexpression in colorectal carcinomas^15^ raises interest about future plans for CYP2W1-based cancer therapy.^6^

Carbonyl reductases (CBRs) and aldo-keto reductases [AKR and Voltage-gated K+ channel beta subunit (KCNAB)] are involved in redox transformations of broad spectrum of carbonyl group-containing xenobiotics, for example, in the transformation of adriamycin to its inactive metabolite adriamycinol.^16–18^ Mammalian AKRs are divided into 3 families AKR1, KCNAB, and AKR7 with 13 identified AKR proteins: AKR1A1 (aldehyde reductase), AKR1B1 and AKR1B10 (aldose reductases), AKR1C1, AKR1C2, AKR1C3, and AKR1C4 (hydroxysteroid dehydrogenases), AKR1D1 (Δ4–3-ketosteroid-5-β-reductase), KCNAB1, KCNAB2, and KCNAB3 (voltage-gated potassium channels), and AKR7A2 and AKR7A3 (aflatoxin reductases).^16^

Taken together, available data in the literature suggest a potential role of drug-metabolizing enzymes in the response of patients to anticancer therapy. However, studies in target tissues of patients are limited and therefore urgently needed for translation of functional data into clinical practice. A comprehensive set of metabolizing enzymes involved in the chemotherapy outcome is, thus, still to be defined.

This study explored gene expression levels of drug-metabolizing enzymes in the posttreatment tissues from breast carcinoma patients treated by neoadjuvant chemotherapy (NACT). Expression profiles were compared with clinical data and with response of the patients to NACT in order to identify putative biomarkers with prognostic and predictive value. Two cohorts of pretreatment patients were then used for comparison and assessment of biological relevance of putative biomarkers on the protein level.

## METHODS

### Materials

Phenol, chloroform, RNase A, proteinase K, ultrapure agarose, and other general chemicals were purchased from Sigma-Aldrich (Prague, Czech Republic). Deoxynucleotides for polymerase chain reaction (PCR) and molecular weight standard for electrophoresis (ΦX174DNA/HaeIII digest) were products of New England Biolabs, Inc (Ipswich, MA). Taq-Purple DNA polymerase and Combi PPP Master Mix for PCR were supplied by Top-Bio s r.o. (Prague). Protein standards for immunoblotting were kindly provided by Prof Paul F. Hollenberg, University of Michigan, Ann Arbor, MI (P450 2B6) and Prof F. Peter Guengerich, Vanderbilt University, Nashville, TN (P450 3A4).

### Patients

Posttreatment tissue samples of human carcinomas of the mammary gland were prospectively obtained from 68 incident breast carcinoma patients diagnosed at the Department of Oncosurgery, Medicon, Prague, during 2006–2010. Patients were treated by NACT based on 5-fluorouracil/adriamycin/cyclophosphamide or 5-fluorouracil/epirubicin/cyclophosphamide and eventually taxanes (for NACT regimens see Table, Supplemental Digital Content 1, http://links.lww.com/MD/A124). Paired adjacent tissue samples without morphological signs of carcinoma (nontumor controls) were available from 43 patients. Collection and pathological processing of tissue samples and retrieval of data was performed as described before.^19^

Pretreatment tissue samples of human carcinomas of the mammary gland were prospectively obtained from 50 incident breast carcinoma patients diagnosed at the Faculty Hospital in Motol, Prague, during 2003–2007. Paired adjacent tissue samples without morphological signs of carcinoma (nontumor controls) were available from 31 patients. Patients were treated by adjuvant chemotherapy and eventually hormonal therapy after surgery (Table, Supplemental Digital Content 1, http://links.lww.com/MD/A124). Collection and pathological processing of tissue samples and retrieval of data was done as described before^20^ (Text, Supplemental Digital Content 2, http://links.lww.com/MD/A124).

For analysis of protein levels of candidate genes, third set was established. Pretreatment tumor tissue samples of human carcinomas of the mammary gland were prospectively obtained from 42 incident histologically verified breast carcinoma patients diagnosed at the Department of Surgery, Hospital Atlas, Zlin, during 2012. Collection and handling of tissue samples and clinical data retrieval adhered to the above-described design (for study flow diagram, see Figure, Supplemental Digital Content 3, http://links.lww.com/MD/A124).

The following data on patients were retrieved from medical records: age, menopausal status, date of diagnosis, personal and family history of cancer, tumor size (pT), lymph node (pN) and distant metastasis (cM), clinical stage, histological type and grade of tumor, expression of estrogen receptor (ER), progesterone receptor (PR), V-ERB-B2 avian erythroblastic leukemia viral oncogene homolog 2 (ERBB2), p53 protein, and Ki-67 protein (for all clinical data, see Table, Supplemental Digital Content 4, http://links.lww.com/MD/A124).

All patients after the primary chemotherapy and surgery were followed for local or distant relapse. Response to NACT was evaluated by Response Evaluation Criteria in Solid Tumors, as described.^21^

All patients were asked to read and sign an informed consent. The study was approved by the Ethical Commission of the National Institute of Public Health in Prague.

### Isolation of Total RNA and cDNA Preparation

Total RNA was isolated from snap frozen tissues using TRIzol Reagent (Invitrogen, Carlsbad, CA). RNA quantity and quality (RIN) was assessed and complementary DNA (cDNA) was synthesized using 0.5 μg of total RNA as described before.^20^ The cDNA was then preamplified using 25 μL of TaqMan PreAmp Master Mix and a pool of 24 specific TaqMan Gene Expression Assays (Life Technologies Corp, Carlsbad; listed in Table, Supplemental Digital Content 5, http://links.lww.com/MD/A124) according to the published procedure.^19^

### Quantitative Real-Time PCR

Quantitative real-time PCR (qPCR) was done and results were evaluated as described before.^19^ Samples from the posttreatment set were preamplified using TaqMan PreAmp Master Mix (Life Technologies Corp). cDNA from the pretreatment set was used for quantification directly without preamplification procedure.

The relative standard curve was generated from 5 log dilutions of 1 nontumor tissue sample (calibrator). Amplification efficiencies for each reference gene (REF) and target gene (TRG) were calculated applying the formula efficiency = 10^–1/slope^ – 1.

*EIF2B1*, *MRPL19*, *IPO8*, and *UBB* were selected as the most stable reference genes for data normalization (Text, Supplemental Digital Content 2, http://links.lww.com/MD/A124). The qPCR study design adhered to the Minimum Information for Publication of Quantitative Real-Time PCR Experiments Guidelines.^22^

Gene expression and clinical data of the evaluation set were submitted to Gene Expression Omnibus repository under accession number GSE56259.

### Immunoblotting in Human Breast Carcinoma Tissues

Tumor tissue samples (n = 42) were stored at −80°C before protein isolation. Samples were grinded using a mortar and pestle and then protein and total RNA was isolated using Allprep DNA/RNA/Protein Mini kit (Qiagen; Hildesheim, Germany) according to the manufacturer's protocol. Total RNA was then used for qPCR of CYP3A4, CBR1, AKR1C1 (Hs04230636_sH), AKR1C2, and AKR7A3 as described above. Protein concentration was determined and immunoblotting was done as previously described.^19^ Briefly, 20 μg of protein was used for separation by sodium dodecyl sulfate polyacrylamide gel electrophoresis (10% gel) and transferred onto 0.2 μm Protran nitrocellulose membrane (Whatman; Kent, UK). Protein standards of CYP2B6, CYP2S1, and CYP3A4 were used in amount of 0.25–1 pmol of purified protein per lane. First, membranes were incubated in blocking solution (Clear Milk Blocking Buffer; Pierce Thermo Scientific, Rockford, IL). Then, membranes were incubated with primary antibodies against CYP2B6 (dilution 1:200; Abgent, San Diego, CA), CYP2S1 (dilution 1 μg IgG/mL), CYP3A4 (dilution 5 μg IgG/mL^23^), AKR1C1 (dilution 1:1000; Aviva Systems Biology, San Diego), AKR1C2 (dilution 1:100; Aviva Systems Biology), AKR7A3 (dilution 1:1000; Genetex, Inc, Irvine, CA), CBR1 (dilution 1:1000; Genetex), or glyceraldehyde phosphate dehydrogenase (GAPDH) (dilution 1:1000; Cell Signaling Technology, Danvers, MA) overnight at 4°C. Membranes were then incubated 2 hours at room temperature with anti-rabbit horseradish-peroxidase-conjugated secondary antibodies (dilution 1:10,000; Sigma Aldrich, Prague, Czech Republic). Protein bands were visualized with an Enhanced Chemiluminescence Detection System (Pierce Biotechnology, Thermo Scientific Pierce Protein Research Products, Rockford, USA) by Carestream Gel Logic 4000 PRO Imaging System (Carestream Health, New Haven, CT). Densitometry was performed using Carestream v5.2 program (Carestream Health) as previously described.^19^

### Cells and Culture Conditions

Human breast carcinoma MDA-MB-231 cell line (without expression of hormonal receptors and ERBB2, ie, triple negative) was purchased from American Type Culture Collection (Manassas, VA). Cells between passages 4 and 40 were used for all experiments. Cell line was authenticated and genomic stability monitored in the fourth and 40th passages by short tandem repeat profiling using PowerPlex ESI 17 Pro System (Promega Corp, Madison, WI). Cells were cultured in basic medium with added 10% fetal bovine serum in a humidified atmosphere of 5% CO_2_ at 37°C. RPMI 1640 containing extra L-glutamine (300 μg/mL), sodium pyruvate (110 μg/mL), 15 mM 4-(2-Hydroxyethyl)piperazine-1-ethanesulfonic acid buffer, penicillin (100 U/mL), and streptomycin (100 μg/mL) was used as a basic medium. The cells were trypsinized by 0.25% trypsin and 0.02% Ethylenediaminetetraacetic acid in phosphate-buffered saline (all chemicals from PANBiotech GmbH, Aidenbach, Germany).

### AKR1C2 siRNA Knockdown

Cells were seeded at 5 × 10^4^ per well (approximately at 70% confluence) of a 24-well plate in culture medium without antibiotics and cultured overnight. Next day culture medium was replaced by transfection mix. For knockdown, a sample of the pools of target small interfering RNA (siRNA) (predesigned, AKR1C2 siRNA, ID: s3991), or positive control (GAPDH siRNA, cat. no.: 4390849) or negative control (cat. no.: 4390846) (all 15 nM/well) in Reduced-Serum Minimal Essential Medium (OPTIMEM) was incubated with Lipofectamine 3000 reagent (all chemicals from Life Technologies), and added to OPTIMEM-conditioned cells in a total volume of 250 μL. After 24 hours incubation, an equal volume of culture medium without antibiotics with 20% fetal bovine serum was added, resulting in final concentrations of 10% fetal bovine serum (500 μL/well). Knockdown efficiencies were determined by qPCR and immunoblotting after 48 hours of growth after addition of culture medium.

### Transfection With pcDNA3.1–CYP3A4 Vector

Cells were seeded at 5 × 10^4^ per well (approximately at 70% confluence) of the 24-well plate in culture medium and cultured overnight. Next day, culture medium was replaced by the fresh complete culture medium (500 μL) including 50 μL of DNA–lipid complex (0.5 μg of plasmid pcDNA 3.1–CYP3A4 or empty plasmid pcDNA 3.1 as negative control; GenScript, Piscataway, NJ). Cells were transfected by mixing with Lipofectamine 3000 reagent according to the instructions of manufacturer (Life Technologies). After 48 hours of transfection, cells were washed and supplemented with the fresh culture medium. Next day, cells were harvested and seeded at approximately 25% confluence onto 24-well plate in culture medium with various concentrations (100, 500, and 1000 μg/mL) of Geneticin (Santa Cruz Biotechnology, Dallas, TX) for selection of geneticin-resistant cells. Selective media were replenished every 3 days and percentage of surviving cells was monitored. After 9 days, 500 μg/mL of geneticin was selected to maintain cell line expressing CYP3A4. CYP3A4 expression was monitored by qPCR and immunoblotting.

### Cell Proliferation Assessment by Flow Cytometry

Cells were seeded at 1 × 10^5^ per well of the 24-well plate and propagated in culture medium. Next day (after 18 hours), culture medium was replaced by the culture medium without drugs (control) or with 100 nM paclitaxel (PCT) or 30 μM adriamycin (LC Laboratories, Woburn, MA). Cells were harvested after 24 hours and fixed in 70% ethanol at 4°C overnight. Fixed cells were washed with phosphate-buffered saline, incubated with 40 μg/mL propidium iodide and 100 μg/mL RNase in phosphate-buffered saline, and cell cycle was analyzed using flow cytometer FACSVersa (Becton, Dickinson and Company, Franklin Lakes, NJ). CYP3A4 and AKR1C2 expression was monitored by qPCR and immunoblotting in parallel samples 48 hours after exposure to drugs.

### Data Analysis

Raw cycle threshold (Ct) data were analyzed by Relative Expression Software Tool (REST) 2009 program (Qiagen, Hildesheim, Germany). Each sample was assayed in duplicate and the mean value was used for calculations. Samples with Ct >40 were treated as missing data. For statistical analyses of associations of transcript levels with clinical data nonparametric tests (Kruskal–Wallis, Mann–Whitney, and Spearman rank) were used. Tested variables were as follows: menopausal status (premenopausal vs postmenopausal), tumor size in millimeter and pT (pT1 vs pT2–4), lymph node metastasis (pN0 vs pN1–3), histological type (ductal vs other invasive breast carcinoma), pathological grade (G1 or G2 vs G3), stage (SI vs SII–SIII), ER, PR, ERBB2, and p53 expression (positive vs negative), Ki-67 expression in percentage of positive tumor cells; and response to NACT (partial pathological response vs stable disease or progression). Samples with complete pathological response after NACT have not been included into the study because of the lack of tumor tissue. Disease-free survival (DFS) was defined as the time elapsed between surgical treatment and disease progression or death from any cause.^20^ Patients lost to follow-up (n = 5 in the posttreatment set) were excluded from the DFS analyses. DFS was evaluated by the Kaplan–Meier method and the log-rank test was used for evaluation of the compared groups of patients. For multivariate analysis, the Cox proportional hazards model was used. *P* values are departures from 2-sided tests. A *P* value of <0.05 was considered statistically significant. Statistical analyses were done using SPSS v16.0 program (SPSS Inc, Chicago, IL). The correction for false discovery rate (FDR) was applied according to Benjamini and Hochberg^24^ and q-values are provided for each comparison.

## RESULTS

### Transcript Levels in Tumors and Nonneoplastic Control Tissues

AKR1A1, AKR1B10, AKR7A3, KCNAB2, and KCNAB3 were significantly overexpressed in tumors compared with nonneoplastic control tissues from the posttreatment set. On the opposite, AKR1C1, AKR1C2, AKR1C3, AKR1C4, and KCNAB1 were significantly downregulated in tumors. CYP1A2, CYP2B6, CYP2D6, CYP2S1, and CYP2W1 were significantly overexpressed whereas CYP2C19, CYP3A4, and CYP3A5 were significantly downregulated in tumors. No significant changes in expression of AKR1B1, AKR1D1, AKR7A2, CBR1, CYP2C8, and CYP2C9 between tumor and control tissues were found. Fold change between tumor and control tissues (mean expression values) with *P* values calculated by REST 2009 are listed in Table 1.

**Table 1:**
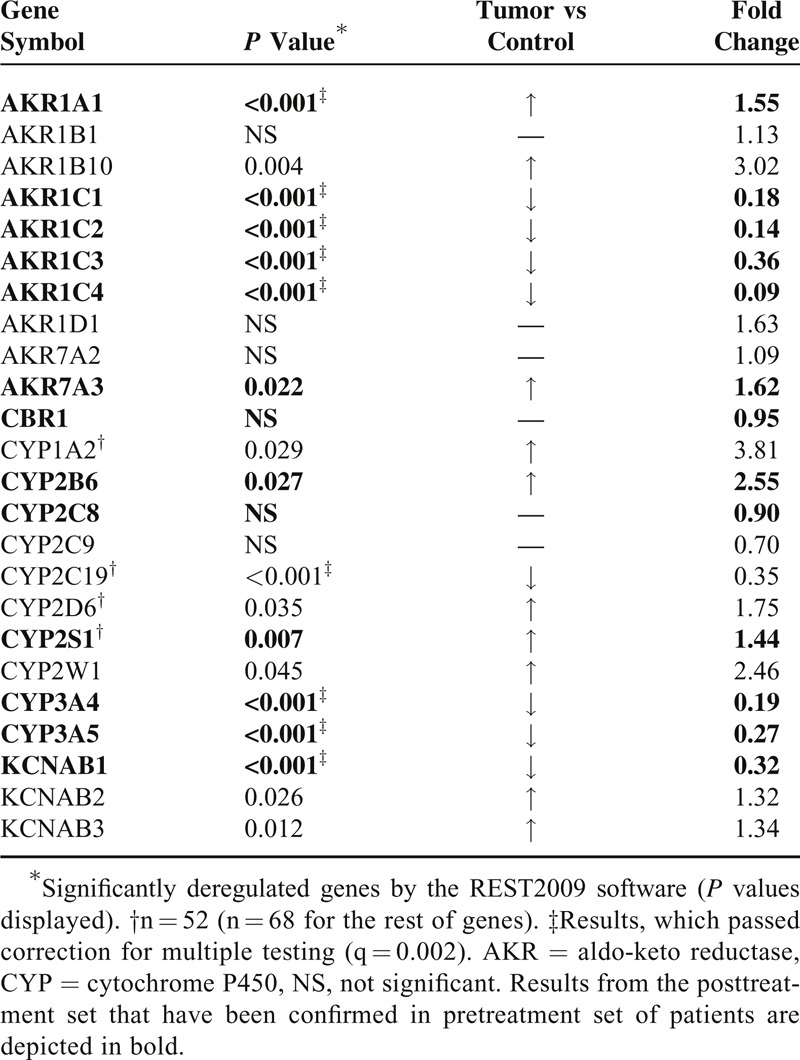
Differences in Transcript Levels Between Tumor and Control Tissues of Breast Carcinoma Patients

### Associations of Transcript Levels With Clinical Data in the Posttreatment Set

Associations of transcript levels of all genes with clinical data were analyzed, but to retain concise style only significant results are reported in Table 2. For this purpose, solely gene expression levels in tumors were evaluated.

**Table 2:**
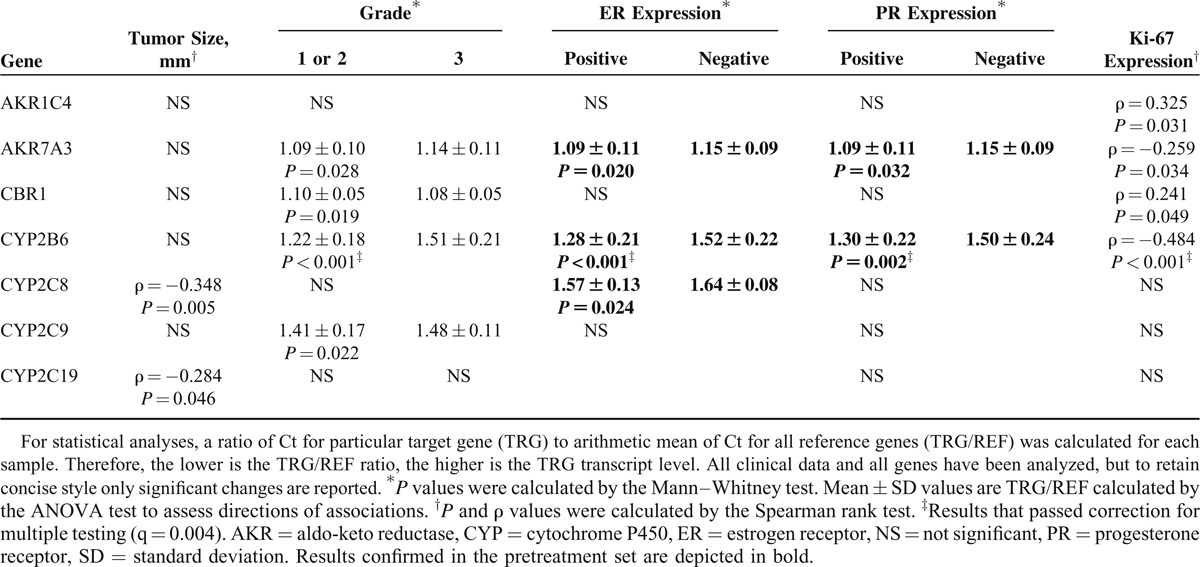
Significant Associations of Intratumoral Transcript Levels With Clinical Data of Patients in the Posttreatment Set

Postmenopausal patients had significantly higher AKR1B10 levels (*P* = 0.026) than the premenopausal patients. Tumor size negatively correlated with CYP2C8 and CYP2C19 levels (Table 2). Patients without lymph node metastasis had significantly higher intratumoral CYP2C9 levels than patients with lymph nodes involved (*P* = 0.049). CBR1 levels were significantly higher in grade 3 (undifferentiated) tumors than in grade 1 or 2 (well or moderately differentiated). On the opposite, AKR7A3, CYP2B6, and CYP2C9 levels were significantly higher in grade 1 or 2 tumors compared with grade 3. CYP3A5 levels were significantly higher in tumors expressing ERBB2 than in ERBB2 negative. Conversely, AKR7A3, CYP2B6, and CYP2C8 levels were significantly higher in ER expressing tumors than in those without ER expression. AKR7A3 and CYP2B6 levels were significantly higher in PR expressing tumors than in those without PR expression. AKR1C4 and CBR1 levels positively, and AKR7A3 and CYP2B6 levels negatively, correlated with Ki-67 protein expression. When correction for multiple testing (Benjamini–Hochberg FDR) was applied, only associations between CYP2B6 levels and grade, and expression of ER, PR, and Ki-67 remained significant (Table 2).

**FIGURE 1:**
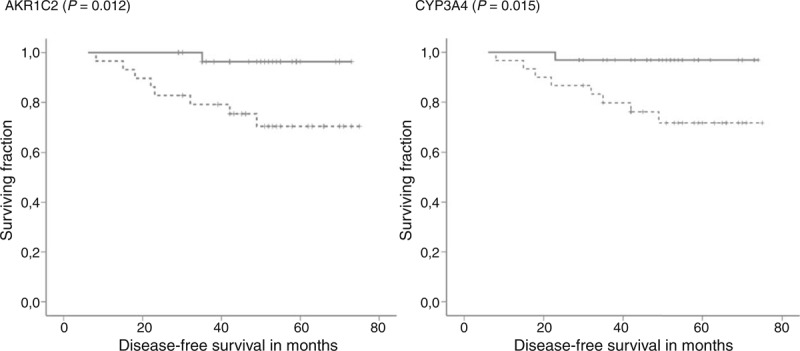
Associations between gene expression levels and DFS of posttreatment patients. Kaplan–Meier survival curves were plotted for patients (n = 65) divided into 2 groups according to the median of transcript levels in tumors. Dashed lines represent the group with lower transcript levels and solid lines represent the group with higher levels than median. Differences between groups were compared using log-rank test. Gene names and significant differences between groups are displayed. All clinical data have been analyzed, but to retain concise style only significant changes are reported. DFS = disease-free survival.

Patients with partial response (responders, n = 38) to NACT had significantly higher intratumoral AKR1C1, AKR1C2, or CYP2W1 transcript levels than patients with stable or progressive disease, that is, nonresponders (n = 24) (1.17 ± 0.15 vs. 1.29 ± 0.13, *P* = 0.003; 1.81 ± 0.19 vs 1.96 ± 0.25, *P* = 0.016; and 1.64 ± 0.14 vs 1.72 ± 0.13, *P* = 0.025; q = 0.004 for all; respectively). Three patients solely treated by hormonal regimens and 2 patients with unknown response were excluded from this analysis. Patients with intratumoral CYP3A4 or AKR1C2 levels higher than median had significantly longer DFS than the remaining patients (n = 63, mean DFS: 71.8 vs 61.5 months, *P* = 0.015, DFS: 71.6 vs 60.9 months, *P* = 0.012, respectively; Figure 1). Multivariate analysis using the Cox regression hazards model with pT, pN, grade, and ER as covariates has confirmed these associations (hazard ratio [HR] = 8.79, 95% confidence interval [CI] = 1.09–70.56, and *P* = 0.041 for CYP3A4 and HR = 9.82, 95% CI = 1.02–94.05, *P* = 0.048 for AKR1C2).

### Associations of Transcript Levels With Clinical Data in the Pretreatment Set

Genes significantly associated with the most important clinical data as grade, expression of hormonal receptors, response to NACT or DFS, and genes strongly deregulated in tumors (significant after correction for multiple testing) in the posttreatment set were included into the study of pretreatment patients. Thus, AKR1A1, AKR1C1, AKR1C2, AKR1C3, AKR1C4, AKR7A3, KCNAB1, CBR1, CYP2B6, CYP2C8, CYP2S1, CYP3A4, and CYP3A5 were further followed in this patient set. As opposed to the posttreatment set no amplification of cDNA was used in the pretreatment set. CYP2C19 and CYP2W1 could not be validated in the pretreatment set owing to gene expression levels below the limit of quantification in samples without preamplification.

As for the posttreatment set, several associations were found (Table 3). However, after correction for multiple testing, only associations between AKR7A3 and expression of ER and those between CYP2B6 and expression of PR, ER, and p53 remained significant. Association between CYP2B6 and expression of hormonal receptors has also previously been observed in the posttreatment set (Table 2). Associations between AKR7A3 or CYP2C8 and expression of ER were also observed in both sets although they did not pass correction for multiple testing in one or both sets. Notable associations between AKR7A3 or CYP2B6 and expression of p53 protein (*P* = 0.006 and *P* < 0.001, respectively; Table 2) could not be compared with the posttreatment set because of the lack of data on p53 expression in this set.

**Table 3:**
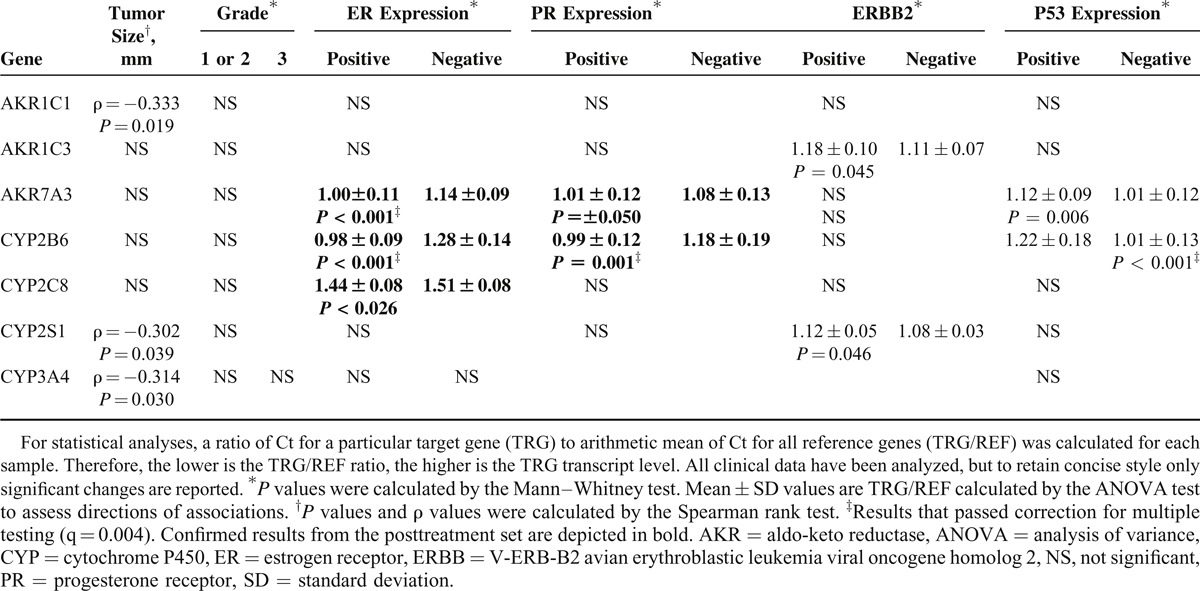
Significant Associations of Intratumoral Transcript Levels With Clinical Data of Patients in the Pretreatment Set

**FIGURE 2:**
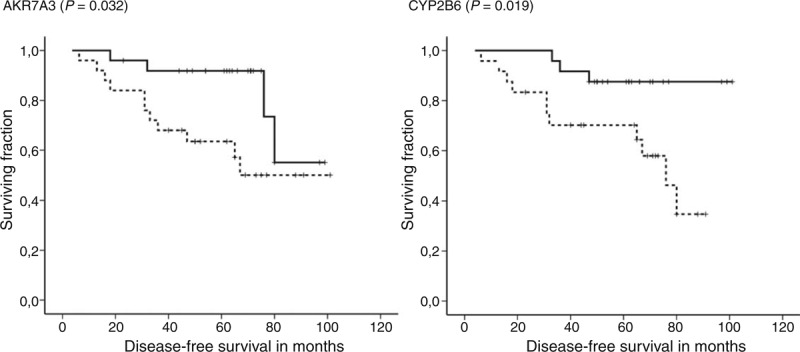
Associations between gene expression levels and DFS of pretreatment patients. Kaplan–Meier survival curves were plotted for patients (n = 50) divided into 2 groups according to the median of transcript levels in tumors. Dashed lines represent the group with lower transcript levels and solid lines represent the group with higher levels than median. Differences between groups were compared using log-rank test. Gene names and significant differences between groups are displayed. All clinical data have been analyzed, but to retain concise style only significant changes are reported. DFS = disease-free survival.

Patients with higher intratumoral AKR7A3 or CYP2B6 levels than median had significantly longer DFS than those with lower levels (n = 50, DFS: 85.3 vs 68.9 months, *P* = 0.032; and DFS: 93.2 vs 64.1 months, *P* = 0.019, respectively; Figure 2). Multivariate analysis using the Cox regression hazards model with pT, pN, grade, and ER as covariates has confirmed association of high AKR7A3 expression with longer DFS (HR = 3.83, 95% CI = 1.03–14.29, and *P* = 0.045), but not that of CYP2B6 with DFS (*P* = 0.083).

The patients from the pretreatment set were also divided into subgroups according to therapy type. Adjuvant chemotherapy-treated patients (n = 25) with higher intratumoral AKR7A3 or CBR1 levels than median had significantly longer DFS than those with lower levels (DFS: 91.1 vs 57.8 months, *P* = 0.040, and DFS: 89.4 vs 58.1 months, *P* = 0.042, respectively; Figure, Supplemental Digital Content 6, http://links.lww.com/MD/A124). Patients treated with hormone (n = 23) with higher intratumoral CYP3A4 or CBR1 levels than median had significantly shorter DFS than those with lower levels (DFS: 52.8 vs 90.4 months, *P* = 0.007, and DFS: all patients with high CBR1 censored, *P* = 0.004, respectively; Figure, Supplemental Digital Content 6, http://links.lww.com/MD/A124). Because of the low numbers of patients in the compared groups, multivariate analysis was not done and the observed trends have to be interpreted with caution.

Transcript levels of CYP2B6 (*P* < 0.001), CYP3A4 (*P* < 0.001), AKR1C1 (*P* < 0.022) AKR1C2 (*P* < 0.001), and AKR7A3 (*P* = 0.001) were significantly lower in posttreatment tumors compared with the pretreatment ones.

### Protein Expression of AKR1C1, AKR1C2, AKR7A3, CYP2B6, CYP2S1, CYP3A4, and CBR1 in Breast Tumors

Putative markers for which transcript levels significantly associated with response to NACT or DFS of the patients were evaluated at the protein level. Expression of AKR1C1, AKR1C2, AKR7A3, CYP3A4, and CBR1 was assessed by immunoblotting in protein lysates from tumor tissue samples of the independent pretreatment set of patients. No protein of the anticipated size corresponding to CYP2B6 or CYP2S1 was detected in the tumor tissues by the commercially available (CYP2B6) or homemade (CYP2S1) antibodies. CYP2B6 and CYP2S1 protein standards were correctly and quite specifically detected by these antibodies using protein standards and human liver microsomes (Figure, Supplemental Digital Content 7, http://links.lww.com/MD/A124). However, we did not observe protein band comigrating with the standard in all inspected tumors. We regularly detected 2 protein bands with molecular weight by approximately 10–15 kg/mol higher than CYP2B6 there. Bands with different molecular weight than the CYP2S1 standard have also been detected in tumors (Figure, Supplemental Digital Content 8, http://links.lww.com/MD/A124).

**FIGURE 3:**
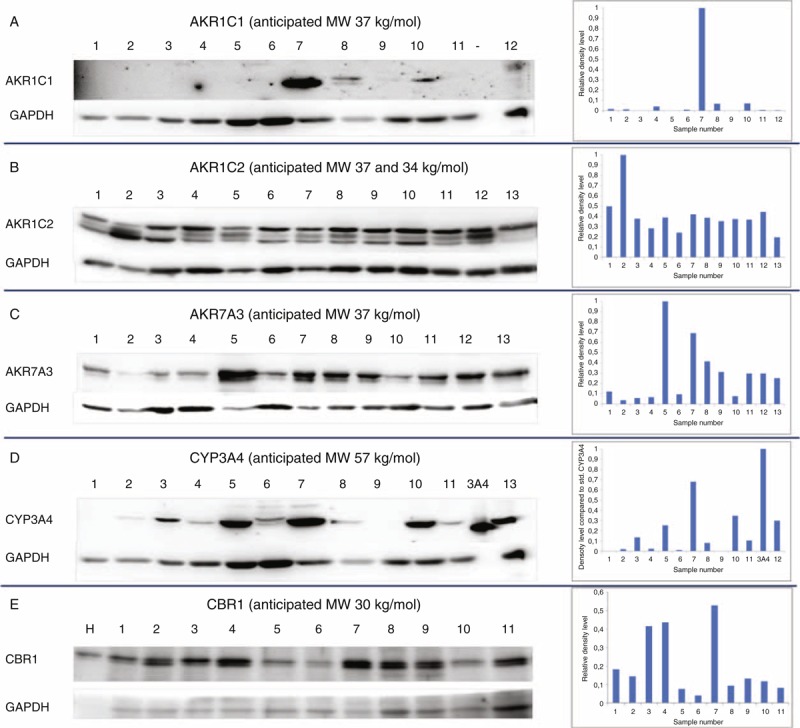
Protein expression of selected candidates in tumors of breast carcinoma patients. Protein expression of (A) AKR1C1, (B) AKR1C2, (C) AKR7A3, (D) CYP3A4, and (E) CBR1 was assessed by immunoblotting (left part) and evaluated by densitometry with normalization to GAPDH (right part) in representative set of breast tumors as described in the “Methods.” Anticipated molecular weight (MW) in kg/mol (in the ±20% range) is presented for each protein according to Human Protein Atlas (http://www.proteinatlas.org). GAPDH = glyceraldehyde phosphate dehydrogenase.

**FIGURE 4:**
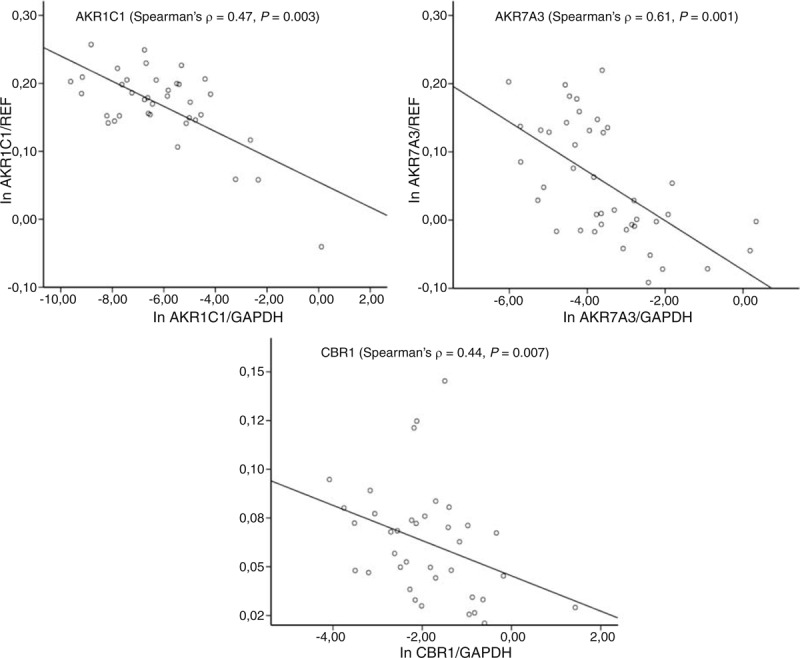
Correlation between protein and transcript levels. Protein levels were analyzed by densitometry with normalization to GAPDH (*X*-axis) and compared with transcript levels normalized to reference genes (*Y*-axis). Normalized protein and transcript levels were logarithmically normalized before comparison. GAPDH = glyceraldehyde phosphate dehydrogenase.

The remaining proteins were well detected and quantified by densitometry. GAPDH expression was used as an internal control for normalization of the results. Purified protein standards (CYP2B6, CYP2S1, and CYP3A4), MT-3 cells lysate (AKR1C2), human liver lysate (AKR1C1 and CBR1), and a pool of tumor samples (AKR7A3) were used as a calibrator for comparison of variability among membranes. Analysis revealed high interindividual variability in expression of all examined proteins (Figure 3). Protein levels of AKR1C1, AKR7A3, and CBR1 significantly correlated with the respective transcript levels assessed by qPCR in the same tumor samples (Spearman ρ = 0.47, *P* = 0.003, Spearman ρ = 0.61, *P* < 0.001, and Spearman ρ = 0.44, *P* = 0.007, respectively) (Figure 4). The protein levels of CYP3A4 and AKR1C2 did not significantly correlate with the respective transcript levels (*P* > 0.05). Three bands recognized by anti-AKR1C2 antibodies in the anticipated molecular weight range were analyzed by densitometry both separately (not shown) and together (Figure 3) with comparable results, that is, lack of correlation with the transcript level.

### Functional Aspects

**FIGURE 5:**
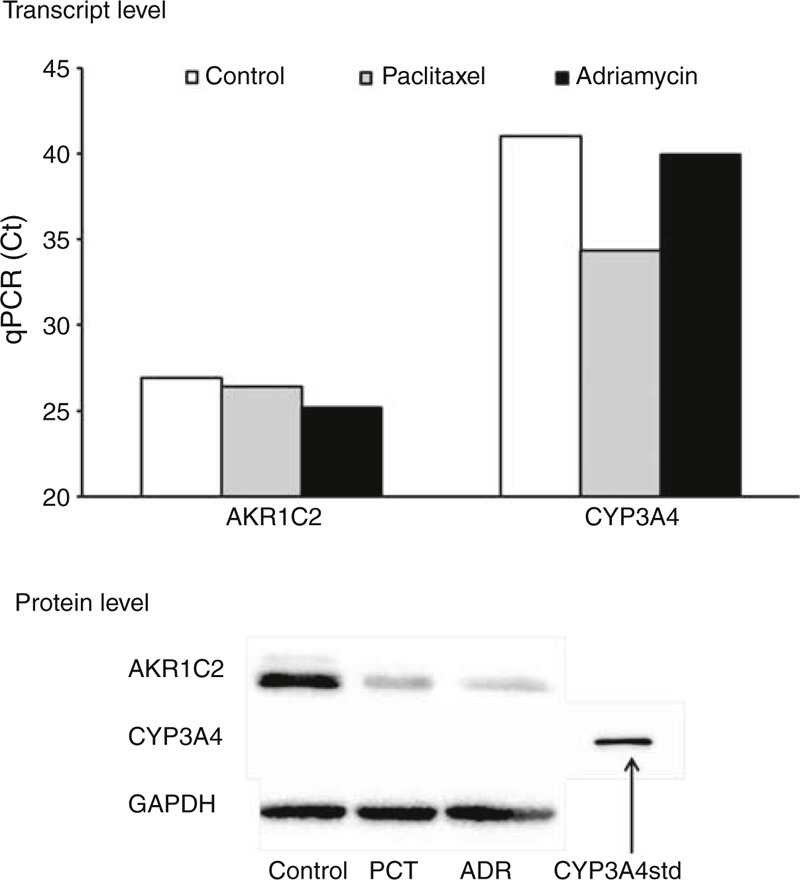
Interaction between PCT or doxorubicin and protein and transcript levels of CYP3A4 and AKR1C2 in vitro. MDA-MB-231 cell line was incubated without (control) or with 100 nM PCT or 30 μM adriamycin (ADR) as described in the “Methods.” qPCR and immunoblotting were done 48 h after the incubation. Ct values from qPCR are presented in the upper part. The higher is the Ct value the lower is the transcript expression. Immunoblots with 10 μg of protein per lane for AKR1C2 and 20 μg of protein or 0.25 pmol of standard for CYP3A4 per lane are presented in the lower part. Two independent experiments were performed with consistent results. Ct = cycle threshold, PCT = paclitaxel, qPCR = quantitative real-time polymerase chain reaction.

AKR1C2 and CYP3A4 were studied in more detail using breast carcinoma model MDA-MB-231 (triple negative) cell line in vitro. In the first experiment, interactions between CYP3A4, AKR1C1, and PCT or adriamycin were addressed. Treatment of the cells with 100 nM PCT resulted in induction of CYP3A4 transcript level, but had no effect on its protein level. AKR1C2 transcript was unaffected, but its protein level was decreased by both 100 nM PCT and 30 μM adriamycin. Adriamycin had no effect on transcript or protein level of CYP3A4 (Figure 5).

**FIGURE 6:**
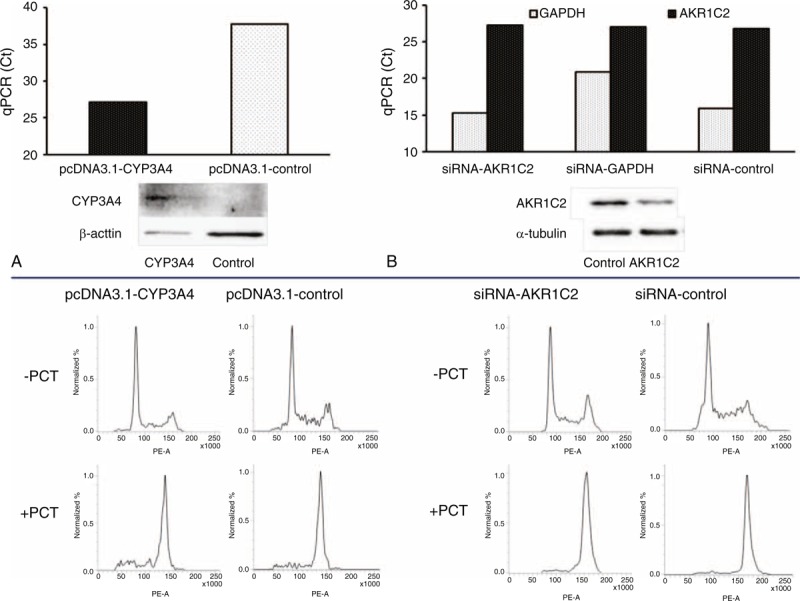
Influence of AKR1C2 silencing and CYP3A4 induction on MDA-MB-231 cell line proliferation after exposure to PCT in vitro. CYP3A4 expression was induced by transfection MDA-MB-231 cell line with the pcDNA3.1-CYP3A4 plasmid (A) and expression of AKR1C2 was decreased by the siRNA against AKR1C2 (B) as described in the “Methods.” Efficiency of cell manipulations was monitored by qPCR and immunoblotting (30 μg of protein per lane for CYP3A4 and 10 μg of protein per lane for AKR1C2). Transcript and protein levels of influenced cells with the respective controls are presented in the upper part. Cells were incubated without (PCT−) or with 100 nM PCT+ for 24 h and then cell proliferation was analyzed using flow cytometry (lower part). Two independent experiments were performed with consistent results. PCT = paclitaxel, siRNA = small interfering RNA.

siRNA-directed knockdown of AKR1C2 expression or pcDNA3.1-CYP3A4 plasmid-mediated upregulation of CYP3A4 expression had no effect on proliferation of MDA-MB-231 cells treated by 100 nM PCT (Figure 6). No effect of 30 μM adriamycin on the MDA-MB-231 proliferation was observed using flow cytometry (results not shown).

## DISCUSSION

Identification of biomarkers with prognostic and predictive value in terms of survival of patients and their response to chemotherapy is a prerequisite step for individualization of cancer treatment.

We studied gene expression of 24 genes coding principal anticancer drug-metabolizing enzymes in tissues from breast carcinoma patients treated by NACT. Our goal was to discover new putative biomarkers with prognostic and predictive value and compare the importance of these biomarkers in 2 groups of patients with different prognoses. In the first phase of analyses, we have identified a number of promising candidates. These candidates were further studied in the set of pretreatment samples and finally protein levels were followed in the third set of samples.

From our observations, we can generalize that the extent of deregulation of gene expression of drug-metabolizing enzymes in tumors of breast carcinoma patients does not strikingly differ between posttreatment and pretreatment patients. However, vast differences between both sets in associations of intratumoral gene expression levels with clinical data of patients observed by this study suggest different prognostic and eventually predictive roles of these particular enzymes.

Taking into consideration the issue of multiple testing, just overexpression of CYP2B6 in tumors expressing hormonal receptors compared with those without such expression is the only universal association typical for both sets. Overexpression of CYP2B6 mRNA in ER-positive breast tumors compared with the normal breast tissue or ER-negative tumors has previously been observed,^25,26^ and therefore, our study validates these results on independent sets of patients. We have observed the association of high CYP2B6 mRNA expression with longer DFS of the pretreatment patients for the first time. Although it has previously been shown that ERs regulate CYP2B6 expression in vitro through direct binding to an estrogen responsive element located in the CYP2B6 promoter,^27^ we have not detected a protein product of the anticipated molecular weight in breast tumors. Thus, we confirmed the lack of CYP2B6 protein in breast tumors reported by others.^28^ In contrast to the published data, we revealed bands of higher molecular weight than the protein standard in all tested samples. The issue of inadequate quality of antibodies is diminished by the fact that the CYP2B6 protein standard was well recognized and the specificity of antibodies was also good. The nature of the protein bands recognized by anti-CYP2B6 antibodies in breast tumors is being studied.

Associations between expression of hormonal receptors and AKR7A3 were observed although they have not passed correction for multiple testing in both sets. Our data support the higher AKR7A3 protein expression in samples from ER-positive breast carcinoma patients as reported previously.^29^ In concordance with the hormonal receptor expression being a factor of more favorable prognosis,^30^ high intratumoral AKR7A3 expression was associated with longer DFS of pretreatment patients in both univariate and multivariate analyses. AKR7A3 protein expression was found in breast tumors for the first time and its high correlation with mRNA levels (*P* < 0.001) demonstrates the biological relevance of AKR7A3 for breast carcinoma. As no other data about the role of AKR7A3 in the prognosis of breast carcinoma patients exist, validation of our findings will be subject of independent follow-up studies.

From other associations found, three may particularly attract further attention. First, responders to NACT had higher intratumoral level of AKR1C2 compared with nonresponders and this association was confirmed by the observed longer DFS in patients with high AKR1C2 level. The association of AKR1C2 with DFS was not observed in the pretreatment patient set suggesting that it may be specific for patients receiving chemotherapy. The present study confirmed the previously observed downregulation of AKR1C2 (and 1C1 and 1C3) in breast tumors compared with nonneoplastic tissues.^31,32^ Our study, however, does not comply with the previously published data showing that AKR1C2 inhibition by 5β-cholanic acid restored sensitivity of adriamycin-resistant human breast adenocarcinoma cell line breast tumor cells in vitro.^33^ We detected AKR1C2 protein in breast carcinomas underlining a potentially functional role of AKR1C2 there. The lack of correlation between AKR1C2 (and CYP3A4) transcript and protein levels observed in the present study may be explained by the use of different normalization controls for qPCR and immunoblotting. The issue of normalization of immunoblotting is a matter of intensive debate.^34^ The influence of posttranscriptional processing and protein stability cannot be ignored as well.

In vitro experiments have shown that PCT and adriamycin reduced AKR1C2 protein expression. However, siRNA-directed knockdown of AKR1C2 had no effect on the proliferation of cells treated by PCT. Taken together, the mechanism of action of AKR1C2 in responders to NACT does not seem to be a result of interactions between AKR1C2 and major drugs used in the breast carcinoma treatment regimens.

Second, patients with high intratumoral CYP3A4 level had significantly longer DFS in the posttreatment set. High CYP3A4 transcript^35^ or protein^36^ levels are predictive for poor response of breast carcinoma patients to docetaxel, which is inactivated by the enzyme. In contrast, CYP3A4 is known as a cyclophosphamide^5^-activating enzyme and from this point of view, the association of high CYP3A4 level with better DFS makes sense in the cyclophosphamide-treated patients (n = 61 in our posttreatment set). In concert with others,^13,28,37^ we also found a striking interindividual variability in intratumoral CYP3A4 protein expression among patients. High CYP3A4 protein expression was previously associated with poor survival of breast carcinoma patients.^13^ CYP3A4 protein level negatively correlated with the transcript levels in our study, which could explain the observed discrepancy of our results with the published data on the prognostic role of CYP3A4. However, this correlation was insignificant (*P* = 0.117) and therefore the observed association between CYP3A4 and DFS must be cautiously interpreted.

PCT transcriptionally activated CYP3A4, but no induction of P450 3A4 protein was detected in vitro by this study. Thus, the functional relevance of such interaction is quite low if any. Adriamycin had no influence on gene or protein expression of CYP3A4 in vitro. Treatment of cells with enhanced CYP3A4 expression by PCT had no effect on the cell proliferation. However, we were able to induce CYP3A4 transcript level to a high extent, but the protein level was poorly induced in the MDA-MB-231 cell line. CYP3A4 is a subject to ubiquitin-dependent proteasomal degradation by the 26S proteasome, a process involving phosphorylation, ubiquitination, and extraction of endoplasmatic reticulum membrane into the cytosol.^38^ Little is known about the nature of these processes in stable cancer cell models as MDA-MB-231. Therefore, for definite answer about the mechanism, in vivo models as mice xenografted with human tumors should be used.

Third, a kind of double-facetted effect was observed for CBR1 in the pretreatment set of patients. A high intratumoral CBR1 level in a chemotherapy-treated subgroup of patients associated with longer DFS, but an opposite effect was found in the hormonal therapy-treated subgroup. These associations have been observed on quite small groups of patients and thus need proper validation in larger cohorts of patients. CBR1 inactivates anthracyclines to the respective alcohols implicated in their cardiotoxicity.^17,39^ Besides the fact that *CBR1* genetic polymorphisms have been shown to influence clearance and exposure levels of adriamycin in breast carcinoma patients,^40^ just one small study observed no significant difference in CBR1 activity between tumor and normal tissues of breast carcinoma patients.^41^ Results of the present study support the recently revealed prognostic significance of decreased CBR1 protein expression (an independent prognostic factor for progression-free and overall survival in multivariate analyses) in endometrial carcinomas.^42^ The same authors previously revealed that suppression of CBR1 expression stimulated cancer cell invasion accompanied with the decrease in E-cadherin expression in uterine cervical squamous cell carcinomas.^43^ We have no explanation for the reversed effect observed in patients treated solely by the hormonal therapy.

We detected CBR1 protein level in all followed tumors and noticed a significant correlation between transcript and protein level. Thus, CBR1 presents another candidate for functional verification in breast carcinoma models.

Additionally, posttreatment patients with high intratumoral CYP2W1 transcript levels responded better to NACT than those with low levels. However, we were unable to validate our results on the nonpreamplified transcript or intratumoral protein levels because of the very low CYP2W1 expression in tumor tissues of the pretreatment set of patients. Thus, CYP2W1 remains an independent biomarker for stages II and III colorectal carcinoma patients,^15^ but not for breast carcinoma. Also, the association between AKR1C1 and response to NACT in the posttreatment set could not be verified on the pretreatment set of patients or on the protein level.

## Conclusions

Associations of AKR1C2, AKR7A3, and CBR1 with prognosis of breast carcinoma patients revealed by this study should be further followed in independent validation and functional studies. The ambiguous roles of CYP2B6 and CYP3A4 noted by this study warrant investigations focused on regulation of their expression and posttranscriptional processing specifically in breast carcinomas.
